# Water Table Management Reduces Tile Nitrate Loss in Continuous Corn and in a Soybean-Corn Rotation

**DOI:** 10.1100/tsw.2001.268

**Published:** 2001-10-25

**Authors:** Craig F. Drury, Chin S. Tan, John D. Gaynor, John W. Daniel Reynolds, Thomas W. Welacky, Thomas O. Oloya

**Affiliations:** Grenhouse &processing Crops Research Centre, Agriculture & Agri-Food Canada, Harrow Ontario, Canada

**Keywords:** nitrate, tile drainage, surface runoff, controlled drainage, subirrigation, water table control, water quality

## Abstract

Water table management systems can be designed to alleviate soil water excesses and deficits, as well as reduce nitrate leaching losses in tile discharge. With this in mind, a standard tile drainage (DR) system was compared over 8 years (1991 to 1999) to a controlled tile drainage/subirrigation (CDS) system on a low-slope (0.05 to 0.1%) Brookston clay loam soil (Typic Argiaquoll) in southwestern Ontario, Canada. In the CDS system, tile discharge was controlled to prevent excessive drainage, and water was pumped back up the tile lines (subirrigation) to replenish the crop root zone during water deficit periods. In the first phase of the study (1991 to 1994), continuous corn (Zea mays, L.) was grown with annual nitrogen (N) fertilizer inputs as per local soil test recommendations. In the second phase (1995 to 1999), a soybean (Glycine max L., Merr.)-corn rotation was used with N fertilizer added only during the two corn years. In Phase 1 when continuous corn was grown, CDS reduced total tile discharge by 26% and total nitrate loss in tile discharge by 55%, compared to DR. In addition, the 4-year flow weighted mean (FWM) nitrate concentration in tile discharge exceeded the Canadian drinking water guideline (10 mg N l) under DR (11.4 mg N l), but not under CDS (7.0 mg N l). In Phase 2 during the soybean-corn rotation, CDS reduced total tile discharge by 38% and total nitrate loss in tile discharge by 66%, relative to DR. The 4-year FWM nitrate concentration during Phase 2 in tile discharge was below the drinking water guideline for both DR (7.3 mg N l) and CDS (4.0 mg N l). During both phases of the experiment, the CDS treatment caused only minor increases in nitrate loss in surface runoff relative to DR. Hence CDS decreased FWM nitrate concentrations, total drainage water loss, and total nitrate loss in tile discharge relative to DR. In addition, soybean-corn rotation reduced FWM nitrate concentrations and total nitrate loss in tile discharge relative to continuous corn. CDS and crop rotations with reduced N fertilizer inputs can thus improve the quality of tile discharge water substantially.

## INTRODUCTION

Tile drainage has enabled farmers to plant earlier in the spring and harvest later in the fall, which has led to increased flexibility in crop type and field operations as well as enhanced crop productivity. In addition, application of nitrogen (N) fertilizers has increased crop yields substantially and is in fact required for economically viable corn production[1]. However it has been known for quite some time that leaching of fertilizer nitrate through the soil profile and into tile drains has contributed to eutrophication of streams and lakes as well as nitrate contamination for surface and groundwater resources[2,3]. Hence an alternative strategy is required to reduce nitrate losses while maintaining crop yields.

Controlled drainage systems have been developed which enable farmers to vary the effective depth of soil profile drainage by installing risers at tile outlets[3,4,5,6,7,8]. The risers on the controlled drainage systems can also be adjusted by the producer to varying depths depending upon the rainfall patterns and time of year so that various field operations can be accommodated[6]. For example, some producers remove or temporarily lower the risers during early spring and late fall periods in order to lower the water table so that planting and harvesting are not delayed.

In regions such as southwestern Ontario, drought conditions frequently occur during mid-July until late August as a result of high evapotranspiration and uneven rainfall distribution[1]. In these regions, irrigation or subirrigation systems are viable means for alleviating water stress as long as an inexpensive source of fresh water is available. Subirrigation involves supplying water directly to the crop root zone by pumping water into tile lines[6]. However, the wetter soil conditions and reduced drainage with a controlled drainage/subirrigation (CDS) system may lead to increased surface runoff when large rainfall events occur[7] or to enhanced nitrate loss through denitrification[9].

The primary objective of this 8-year study is to determine the effectiveness of a CDS system for reducing tile flow, decreasing nitrate loss, and increasing water and nitrate storage in the soil of a systematically tile-drained field in southwestern Ontario. The influence of crop rotation on nitrate loss was also evaluated by comparing 3.5 years of continuous corn production, which requires annual inputs of N fertilizer, to 4 years of a soybean-corn rotation in which fertilizer N is applied only during the corn years.

## EXPERIMENTAL METHOD/PROCEDURES

### Treatments and Experimental Design

This study was initiated on a Brookston clay loam (fine-loamy, mixed, mesic Typic Argiaquoll) in 1991. There were 310 g $kg^{-1}$ clay, 388 g $kg^{-1}$ silt, and 302 g $kg^{-1}$ sand in the top 30 cm. The average saturated hydraulic conductivity ($k_{sat}$) was 4.7 cm $day^{-1}$ in the 0- to 1.2-m depth, 0.06 cm $day^{-1}$ in the 2- to 3.5-m depth, and 0.02 cm $day^{-1}$ below the 3.5-m depth. Hence the soil was virtually impermeable below 2 m. There were two phases of 3.5 and 4 years each (i.e., Phase 1 from 1991 to 1994 and Phase 2 from 1995 to 1999) evaluated in this study. In both phases, two water management systems were compared: unrestricted tile drainage (DR), which is typical of the systematic drainage systems in southwestern Ontario; and CDS where tile discharge is controlled to prevent excessive drainage and where water is pumped back up the tile lines during water deficit periods to subirrigate the crop root zone.

For Phase 1, corn (*Zea mays* L., Pioneer corn hybrid 3573) was seeded at a rate of 65,000 seeds $ha^{-1}$ in 75-cm-wide rows with a Kinze four-row planter (Kinze Manufacturing Company Inc., Williamsburg, IA). Starter fertilizer 8-32-16 ($N$-$P_2O_5$-$K_2O$) was banded beside the seed at a rate of 132 kg $ha^{-1}$. Urea (46-0-0) was applied with a brush applicator at the six-leaf stage. In 1991, urea was applied at 115 kg N $ha^{-1}$. This rate was adjusted from an average rate for this region of 170 kg N $ha^{-1}$ to account for N released from the alfalfa plowed down in the fall of 1990. A conservation tillage system using a modified chisel plow (Soil Saver) was used in the fall after corn harvest. In 1992 to 1994, sidedressed urea was applied at an average rate of 167 kg N $ha^{-1}$, based on the average $NO_3^-$ soil test of soil samples collected on the day of planting. Hence the total N application for Phase 1 was 658 kg N $ha^{-1}$, which includes the starter fertilizer and sidedress N application. Weed control was provided by atrazine (1.1 kg a.i. $ha^{-1}$), metribuzin (0.5 kg $ha^{-1}$), and metolachlor (1.68 kg $ha^{-1}$) applied in a band over the corn row immediately after planting. There were two replicates for each treatment in Phase 1.

Phase 2 involved a soybean-corn rotation with soybeans grown in 1995 and 1997, and corn grown in 1996 and 1998. Corn received a starter fertilizer application of 142 kg $ha^{-1}$ 18-46-0 at planting (based on soil test results) and a sidedress application of 125 kg N $ha^{-1}$ as urea at the six-leaf stage. Hence the 4-year total fertilizer N input during Phase 2 was 300 kg N $ha^{-1}$, which includes both the initial starter fertilizer as well as the sidedress N application.

Soybean (Asgrow A2615) was planted at 97.4 kg $ha^{-1}$ with a Great Plains (Great Plains Manufacturing Inc., Assaria, KS) no-till drill in 20.3-cm-wide rows on May 23, 1995 and June 12, 1997. Corn (Pioneer 3515) was seeded May 28, 1996 and May 21, 1998 at a rate of 79,700 seeds $ha^{-1}$ in 75-cm-wide rows with a Kinze four-row planter. The corn variety was changed in Phase 2 as the variety used in Phase 1 was no longer available. Weed control in soybean was provided by metribuzin (0.56 kg a.i. $ha^{-1}$) and metolachlor (1.92 kg a.i. $ha^{-1}$) applied pre-emergence and broadcast in 1995 and 1997. Weed control in corn was provided by atrazine (1.1 kg a.i. $ha^{-1}$), metribuzin (0.5 kg $ha^{-1}$), and metolachlor (1.68 kg $ha^{-1}$) applied pre-emergence broadcast. No tillage was used with the only soil disturbance being that associated with the planting and fertilizer application. In Phase 2, the number of replicates was increased from two to three per treatment.

### Field Layout and Installation

Details of the field layout and installation have been reported previously[6,7]. There were 16 plots in the experiment with the results from 4 plots (two treatments and two replicates) reported in Phase 1 (1991 to 1994) and from 6 plots (two treatments and three replicates) reported in Phase 2 (1995 to 1999). Each plot was 15 m wide × 67 m long (1005-$m^2$ area) with 0.05 to 0.1% slope. There were two tile drains 104 mm in diameter parallel to the length in each plot. Hydrological isolation among plots was achieved by (1) installing double-layer, 4-mil-thick, plastic “curtain walls” from the surface to a 1.2-m depth along the plot edges, (2) separating each plot with a 7.5 m wide × 67 m long buffer area with a single tile drain, and (3) encircling each plot with a 30-cm-high berm to contain surface runoff.

In the CDS plots, risers were used for subirrigation and controlled drainage during the growing season, and for controlled drainage during fall, winter, and spring[6]. Risers installed on tile drain outlets of the CDS treatments effectively raised the tile depth from 0.6 to 0.3 m below the soil surface. To ensure that planting and harvesting operations would not be impeded by wet soil conditions, the risers were then removed in late April in preparation for a mid-May planting, reinstalled after planting in early June, removed again before harvest in mid-October, and reinstalled after harvest in mid-November. Subsurface irrigation on the CDS treatments was initiated when the water level in the risers fell below 30 cm. Generally, the CDS treatment received subirrigation from mid-June until the end of August. This was accomplished by pumping water from a storage pond to the riser reservoir via an underground polyethylene pipe 50 mm in diameter. Water meters located at the control structures record the total volume of irrigation water delivered to each plot (Table 1).

Within each plot, two existing tile drains (104 mm in diameter at 7.5-m spacing and 0.6-m depth) were connected to collect subsurface drainage. The drainage was routed to a central instrumentation building at the northeast corner of the experimental field via 104-mm corrugated, nonperforated drain pipes. Each plot also has a surface catch basin 0.5 m in diameter at its east boundary to collect the surface runoff and direct it to the central instrumentation building through a second set of underground, nonperforated drain pipes. The 6 × 8 m instrumentation building is equipped to allow year-round data collection and includes an automatic backup generator to ensure uninterrupted data collection during power outages.

## Surface and Tile Flow Measurements

Surface runoff, subirrigation, and tile drainage volumes were recorded continuously from May 1991 to October 31, 1994 (Phase 1) and from May 1, 1995 until April 30, 1999 (Phase 2) by Neptune T-10 water meters[10]. Surface and tile drainage water from each plot is collected in polyethylene sumps (500-mm diameter × 750 mm deep) located within the instrumentation building. Each sump is equipped with an electrical, float-activated, effluent pump. Surface runoff and tile drainage from each individual plot was pumped through water meters to an outlet drain. Each water meter records flow volumes mechanically as well as from analog and digital pulse signals. A multichannel datalogger utilizes the analog signal of the water meters to monitor, measure, and store water volumes on a continuous basis[10]. Every 24 h, the data stored in the dataloggers are automatically transmitted over a telephone line to a computer at the main research facility 32 km away.

**TABLE 1**
**Precipitation, Subsurface Irrigation Volumes for the CDS Treatment, Tile Discharge, FWM Nitrate Concentrations, and Cumulative Nitrate Loss in Tile Discharge for the DR and CDS Treatments during Phase 1 (Continuous Corn) and Phase 2 (Soybean-Corn Rotation)**

| Year | Precipitation (mm) | Subsurface Irrigation (mm) | Tile Discharge (mm) | | FWM Nitrate Concentration in Tile Discharge (mg N $l^{-1}$) | | Cumulative Nitrate Loss in Tile Discharge (kg N $ha^{-1}$) | |
|---|---|---|---|---|---|---|---|---|
| | | | DR | CDS | DR | CDS | DR | CDS |
| Phase 1 | | | | | | | | |
| 1991-1992* | 763 | 89 (20)** | 257 (26) | 212 (1) | 22.8 (1.8) | 12.7 (0.5) | 58.2 (1.3) | 26.8 (1.3) |
| 1992-1993 | 954 | 7 (2) | 285 (2) | 166 (42) | 4.0 (0.1) | 2.3 (0.5) | 11.3 (0.2) | 3.6 (0.2) |
| 1993-1994 | 728 | 121 (13) | 120 (10) | 113 (2) | 5.3 (0.3) | 3.1 (0.3) | 6.3 (0.1) | 3.5 (0.3) |
| 1994 | 356 | 63 (4) | 15 (2) | 12 (0) | 9.3 (1.3) | 7.8 (0.4) | 1.4 (0.1) | 1.0 (0.1) |
| Total: 1991-1994 | 2801 | 280 | 677 (35) | 503 (46) | 11.4 (0.4) | 7.0 (0.4) | 77.2 (1.5) | 34.9 (1.2) |
| Phase 2 | | | | | | | | |
| 1995-1996 | 633 | 103 (16) | 119 (9) | 70 (1) | 7.7 (0.6) | 3.3 (0.2) | 9.1 (0.1) | 2.3 (0.1) |
| 1996-1997 | 744 | 104 (11) | 315 (18) | 178 (14) | 9.3 (0.4) | 5.6 (0.2) | 29.2 (0.4) | 10.0 (1.0) |
| 1997-1998 | 800 | 48 (9) | 249 (26) | 142 (37) | 4.8 (0.5) | 2.3 (0.2) | 11.7 (0.2) | 3.3 (1.1) |
| 1998-1999 | 625 | 88 (23) | 113 (16) | 107 (7) | 6.9 (0.6) | 3.8 (0.3) | 7.6 (0.5) | 4.0 (0.4) |
| Total: 1995-1999 | 2802 | 343 | 796 (64) | 496 (55) | 7.3 (0.5) | 4.0 (0.1) | 57.6 (0.9) | 19.7 (2.4) |

* The measurement periods were based on cropping season (i.e., from May 1 until April 30) except for 1994 where the measurement period was from May 1, 1994 until October 31, 1994. The totals for 1991 to 1994 were for a 3.5-year period (i.e., from May 1, 1991 until October 31, 1994) whereas the totals for 1995 to 1999 were for a 4-year period (from May 1, 1995 until April 30, 1999).

** Numbers in parenthesis are standard error (n = 2 for 1991 to 1994, n = 3 for 1995 to 1999).

## Water Sampling and $NO_3^-$ Analyses

Samples of surface runoff and tile drainage water were collected automatically with autosamplers (CALYPSO 2000S, Buhler Gmbh & Co) located in the instrumentation building. The autosampler is activated by digital signals from the water flow meter. Sample collection was based on flow volume with collections every 500 to 3000 l depending upon the time of year and expected runoff volumes. After a runoff-producing event, the water samples were quickly removed from the autosamplers and stored at 4°C until they were analysed for $NO_3^-$ concentration. Sample analysis included filtering through a 0.45 mm filter (Gelman GN-6, Gelman Sciences, Ann Arbor, MI) and analysis on a TRAACS 800 autoanalyzer (Bran + Leubbe, Buffalo Grove, IL) for $NO_3^-$ using the cadmium reduction method[11]. Flow weighted mean (FWM) $NO_3^-$ concentrations were calculated for 1-year and either 3.5- or 4-year periods by dividing cumulative nitrate loss by cumulative flow[12].

## Statistical Analysis

Analysis of variance was conducted on the flow volumes, $NO_3^-$ concentrations, and cumulative $NO_3^-$ losses[13]. A randomized complete block design was used with two replicates in Phase 1 and three replicates in Phase 2. Unless otherwise noted, statistical significance is reported at the 0.05 probability level.

## RESULTS AND DISCUSSION

The cumulative tile drainage volumes were substantially greater than the corresponding surface runoff volumes in Phase 1 especially after March 1992 (Fig. 1). The water volumes followed a step-wise pattern with large tile flows occurring during the late winter to spring periods (December to April). In southwestern Ontario, the colder air temperatures during the winter and spring period and absence of growing crops reduces evapotranspiration and hence more water is lost through tile drainage and surface runoff, as expected[6,14]. In Phase 1, there was a significant decrease in tile flow (26%) with CDS compared to DR, and a corresponding increase in the surface runoff. It should be noted, however, that even though the water inputs were greater with the CDS system (i.e., precipitation and subirrigation) as compared to DR (i.e., precipitation only), there was proportionally less tile drainage with the CDS system.

In Phase 2, tile flow also followed a step-wise pattern over the 4 years, and CDS reduced tile flow considerably (38%) compared to the DR treatment (Fig. 1). The surface runoff was increased with the CDS system, however the decrease in tile flow was greater than the increase in surface runoff. In general, total tile drainage volume (Table 1) with the DR treatment in Phase 2 (796 mm) was somewhat greater than the drainage volumes in Phase 1 (677 mm). This is because in Phase 1, tile water in 1994 was only measured over a 6-month cropping period (May 1 to October 31, 1994)

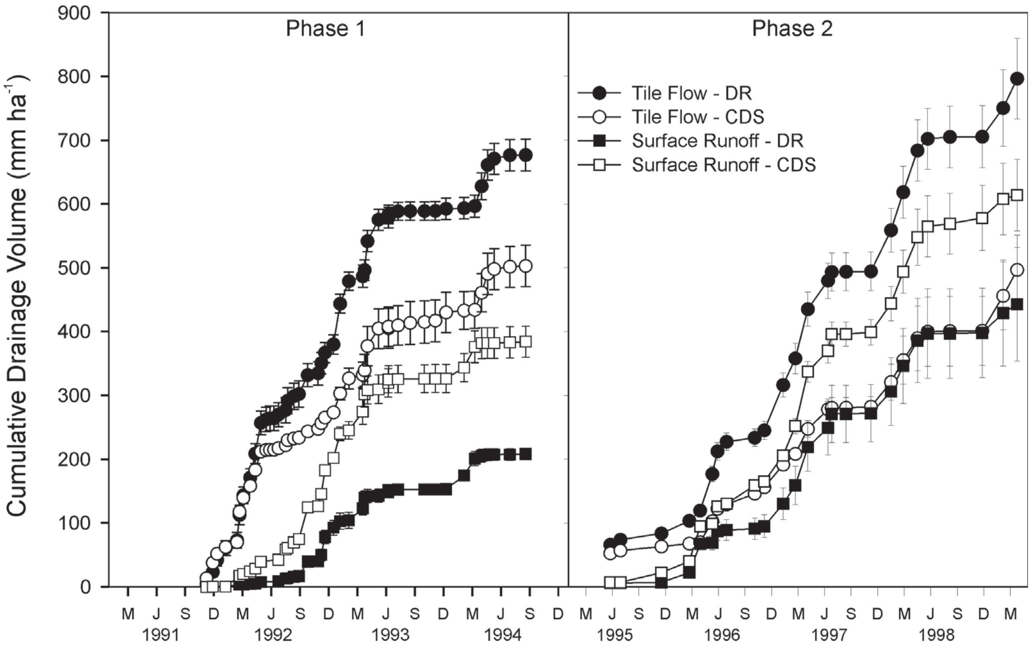

**FIGURE 1.** Cumulative tile flow and surface runoff from DR and CDS with continuous corn (Phase 1) and a soybean-corn rotation (Phase 2). Error bars are standard error (n = 2 for Phase 1 and n = 3 for Phase 2).

and produced extremely low tile drainage volumes (Table 1). The tile discharge volumes varied considerably among years as a result of differences in precipitation (Table 1), crop growth, and evapotranspiration.

Surface runoff volumes were greater in Phase 2 than Phase 1, which is likely due to the lower residue associated with soybean relative to corn (Fig. 1 and Table 2). Differences in soil water storage, evapotranspiration, and rainfall distribution may have contributed as well, although the total amount of precipitation for Phases 1 and 2 was similar (2801 mm for Phase 1 and 2802 mm for Phase 2).

The nitrate concentrations in the Phase 1 tile drainage water in the fall of 1991 and spring of 1992 were much greater than the drinking water guideline value 10 mg N $l^{-1}$ (Fig. 2). These high concentrations were the combined result of mineralization of alfalfa, which was plowed down in the fall of 1990, plus the N fertilizer applied in 1991. Higher nitrate concentrations were also observed following sidedress N application (the time of sidedress application was indicated by arrows at the top of the graph). During Phase 1, the FWM nitrate concentration of tile drainage water from the DR treatment (11.4 mg N $l^{-1}$) was greater than that from corresponding CDS treatment (7.0 mg N $l^{-1}$), amounting to a 39% reduction for CDS (Table 1).

The nitrate concentrations in tile drainage water in Phase 2 were comparatively lower than in Phase 1 as a result of lower N inputs with a soybean-corn rotation than with continuous corn (Fig. 2 and Table 1). The FWM nitrate concentrations over the 4-year period in Phase 2 were 7.3 mg N $l^{-1}$ for the DR treatment vs. 4.0 mg N $l^{-1}$ for the CDS treatment (i.e., a 45% reduction with CDS). Further, the DR treatment had a 36% lower FWM nitrate concentration in Phase 2 than Phase 1, which likely reflects the fact that about 50% less fertilizer N was added in Phase 2 (soybean-corn rotation) than in Phase 1 (continuous corn). Similarly, the FWM nitrate concentration for the CDS treatment was 43% lower in Phase 2 than Phase 1.

The FWM nitrate concentration in surface runoff was very small with averages of 1.8 to 1.9 mg N $l^{-1}$ in Phase 1 and 2.3 to 2.5 mg N $l^{-1}$ in Phase 2 (Table 2). The only time when the drinking water guideline value was exceeded in surface runoff was 1994 (May 1 to October 31) when the FWM nitrate concentration from the CDS treatment was 22.9 mg N $l^{-1}$. The CDS flow for this 6-month period was very low at 2 mm, however, which was <1% of the total Phase 1 flow reported for the CDS treatment (384 mm). Thus, even though CDS produced greater surface runoff than DR, the total $NO_3^-$-N loss in runoff was small from both treatments (Table 2).

**TABLE 2**
**Surface Runoff, FWM Nitrate Concentrations and Cumulative Nitrate Loss in Surface Runoff for the DR and CDS Treatments during Phase 1 (Continuous Corn) and Phase 2 (Soybean-Corn Rotation)**

| Year | Subsurface Runoff (mm) | | FWM Nitrate Concentration in Surface Runoff (mg N $l^{-1}$) | | Cumulative Nitrate Loss in Surface Runoff (kg N $ha^{-1}$) | |
|---|---|---|---|---|---|---|
| | DR | CDS | DR | CDS | DR | CDS |
| Phase 1 | | | | | | |
| 1991-1992 | 7 (4)* | 40 (9) | 6.9 (0.1) | 2.3 (0.4) | 0.5 (0.3) | 1.0 (0.4) |
| 1992-1993 | 135 (19) | 269 (22) | 1.4 (0.1) | 1.5 (<0.1) | 2.0 (0.4) | 4.0 (0.3) |
| 1993-1994 | 65 (1) | 73 (3) | 2.3 (<0.1) | 2.2 (<0.1) | 1.5 (<0.1) | 1.6 (0.1) |
| 1994 | 1 (1) | 2 (2) | 3.8 (0.3) | 22.9 (6.8) | <0.1 (<0.1) | 0.5 (0.2) |
| Total: 1991-1994 | 208 (12) | 384 (35) | 1.9 (0.1) | 1.8 (0.1) | 4.0 (<0.1) | 7.1 (0.9) |
| Phase 2 | | | | | | |
| 1995-1996 | 67 (9) | 95 (3) | 3.1 (0.6) | 1.9 (0.2) | 2.2 (0.7) | 1.8 (0.1) |
| 1996-1997 | 152 (28) | 242 (13) | 2.6 (0.6) | 2.8 (0.5) | 4.3 (1.7) | 6.8 (1.3) |
| 1997-1998 | 167 (29) | 211 (29) | 2.3 (0.5) | 1.4 (0.1) | 4.1 (1.6) | 3.0 (0.4) |
| 1998-1999 | 57 (23) | 66 (12) | 2.4 (0.5) | 3.9 (0.8) | 1.4 (0.7) | 2.7 (0.9) |
| Total: 1995-1999 | 443 (89) | 614 (56) | 2.5 (0.5) | 2.3 (0.3) | 12.0 (4.7) | 14.3 (2.2) |

* The measurement periods were based on cropping season (i.e., from May 1 until April 30) except for 1994 where the measurement period was from May 1, 1994 until October 31, 1994. The totals for 1991 to 1994 were for a 3.5-year period (i.e., from May 1, 1991 until October 31, 1994) whereas the totals for 1995 to 1999 were for a 4-year period (from May 1, 1995 until April 30, 1999).

** Numbers in parenthesis are standard error (n = 2 for 1991 to 1994, n = 3 for 1995 to 1999).

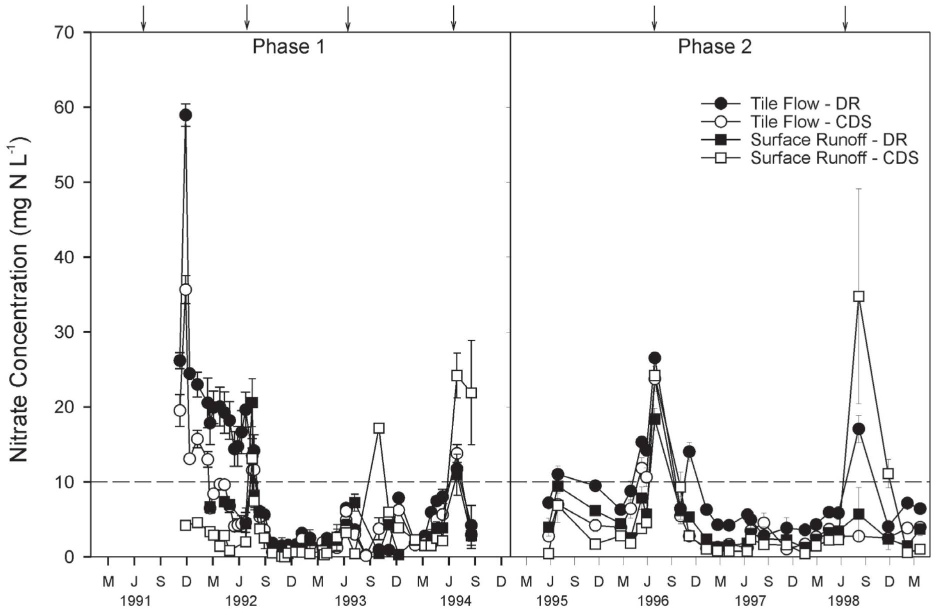

**FIGURE 2.** Nitrate concentration in tile flow and surface runoff from tile DR and CDS with continuous corn (Phase 1) and a soybean-corn rotation (Phase 2). The arrows refer to the times when sidedress N was applied to corn and the dashed line is the water quality guideline value of 10 mg N $l^{-1}$. Error bars are standard error (n = 2 for Phase 1 and n = 3 for Phase 2).

The total $NO_3^-$-N loss in tile drainage water was consistently greater from the DR treatment than from the CDS treatment as a result of the greater flow volumes and higher nitrate concentrations in tile drainage water (Table 1 and Figs. 1 to 3). In particular, the total $NO_3^-$-N loss for Phase 1 was 77.2 kg N $ha^{-1}$ for the DR treatment compared to 34.9 kg N $l^{-1}$ for the CDS treatment, which is a 55% reduction for CDS (Table 1). Similar results have been found in other studies[3,6,8,14,15]. The cumulative $NO_3^-$-N lost through tile drainage also varied among years as a result of the varying amounts of precipitation (Table 1). The greatest $NO_3^-$-N loss occurred in 1991 to 1992 with 58.2 kg N $ha^{-1}$ lost from the DR treatment vs. 26.8 kg N $ha^{-1}$ lost from the CDS treatment. This occurred because of the higher N concentration in tile drainage water stemming from mineralization and nitrification and leaching of N from alfalfa that was plowed down in the fall of 1990.

Over the 4-year Phase 2 period, the CDS treatment reduced $NO_3^-$-N losses by an average of 66% relative to DR (19.7 kg N $ha^{-1}$ from CDS; 57.6 kg N $ha^{-1}$ from DR) (Table 1). The reductions in $NO_3^-$-N loss with the CDS treatment were also apparent in each year of Phase 2, ranging from 47% in 1998 to 1999 to 75% in 1995 to 1996. The annual $NO_3^-$-N loss in tile drainage from the DR treatment was greatest (29.2 kg N $ha^{-1}$) in 1996 to 1997 compared to all other years in Phase 2. This was a combined result of both higher drainage volume and higher nitrate concentration in tile drainage water for that year. It should also be noted that sidedress N fertilizer was added to corn on June 26, 1996 (Fig. 3).

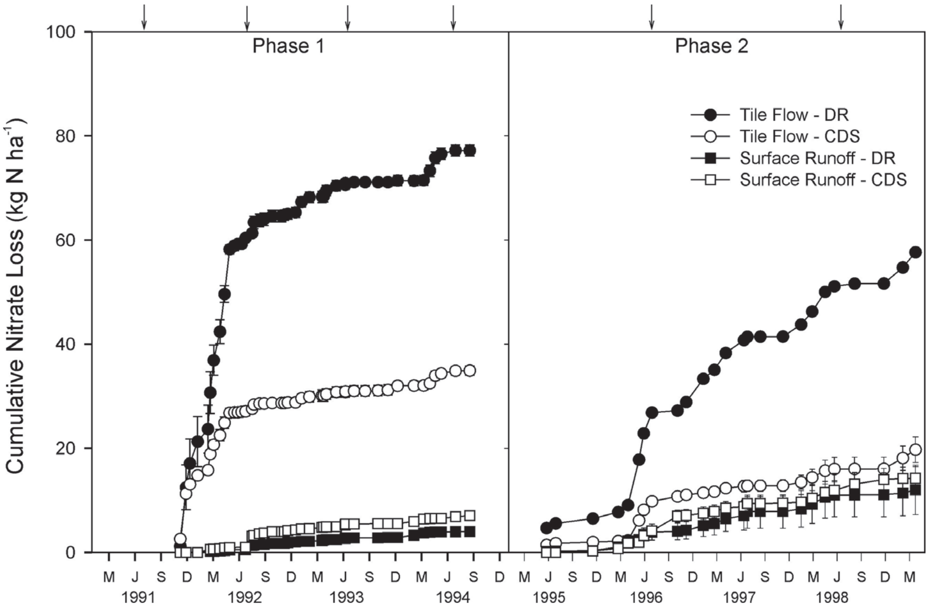

**FIGURE 3.** Nitrate loss in tile flow and surface runoff from DR and CDS with continuous corn (Phase 1) and a soybean-corn rotation (Phase 2). The arrows refer to the times when sidedress N was applied to corn. Error bars are standard error (n = 2 for Phase 1 and n = 3 for Phase 2).

The total nitrate loss through surface runoff in Phase 1 was 7.1 kg N $ha^{-1}$ for CDS and 4.0 kg N $ha^{-1}$ for DR treatment. Surface runoff losses were two- to three-times greater in Phase 2 with 14.3 kg N $ha^{-1}$ lost from the CDS treatment and 12.0 kg N $ha^{-1}$ lost from the DR treatment. This occurred primarily because there was 1.6- to 2.1-times greater surface runoff in Phase 2 than Phase 1. Although there was 2 to 3 kg N $ha^{-1}$ greater $NO_3^-$-N loss in surface runoff with the CDS in these two phases compared to the DR treatment, this small increase was more than compensated by the 37.9 to 42.3 kg N $ha^{-1}$ lower nitrate loss from CDS than from DR in tile drainage.

## CONCLUSIONS

CDS effectively decreased tile flow volume, nitrate concentration in tile drainage water, and total amount of nitrate lost through the tile discharge relative to DR. Under CDS, the FWM nitrate concentrations of tile drainage water were less than the drinking water guideline (10 mg N $l^{-1}$) in both phases of the study (i.e., 7.0 mg N $l^{-1}$ in Phase 1 and 4.0 mg N $l^{-1}$ in Phase 2) (Table 1). In contrast, the guideline value was exceeded under the DR treatment for Phase 1 (continuous corn) at 11.4 mg N $l^{-1}$, and it approached the guideline value in 1996 to 1997 of Phase 2 (9.3 mg N $l^{-1}$). It should be noted that fertilized corn was grown in the 1996 field season. The FWM nitrate concentration was well within the guidelines under both DR and CDS for both soybean years (1995 to 1996 and 1997 to 1998) in Phase 2 with a range between 2.3 to 3.3 mg N $l^{-1}$ for CDS and 4.8 to 7.7 mg N $l^{-1}$ for DR (Table 1). The FWM nitrate concentration in surface runoff was very low for Phase 1 (1.8 to 1.9 mg N $l^{-1}$) and Phase 2 (2.3 to 2.5 mg N $l^{-1}$) (Table 2). Although nitrate losses in surface runoff were increased with the CDS treatment, these were minor compared to the amount of N lost through tile drainage with the DR treatment. In Phase 2, crop rotation involving soybeans also contributed to lower $NO_3^-$-N losses through tile drainage primarily because no N fertilizer was added in the soybean phase of the rotation.

## ACKNOWLEDGEMENTS

The Great Lakes Water Quality Preservation Fund provided funding for Phase 1 of this study. Appreciation is also expressed for expert technical assistance of K. Rinas, M. Soultani, D. MacTavish, V. Bernyk, W. Calder, G. Stasko, S. Mannell, A. Szabo, and M. Bissonnette.

| | | |
|---|---|---|
| Received: | July | 16, 2001 |
| Revised: | September | 24, 2001 |
| Accepted: | October | 3, 2001 |
| Published: | October | 25, 2001 |
